# Processing of corn-based dog foods through pelleting, baking and extrusion and their effect on apparent total tract digestibility and colonic health of adult dogs

**DOI:** 10.1093/jas/skae067

**Published:** 2024-03-30

**Authors:** Isabella Corsato Alvarenga, Ryan Lierz, Youhan Chen, Andrea Lu, Nanyan Lu, Charles G Aldrich

**Affiliations:** International Flavors & Fragrances/Danisco, New Century, Kansas 66031, USA; The J.M. Smucker Company, Orrville, Ohio 44667, USA; Department of Grain Science and Industry, Kansas State University, Manhattan, Kansas 60523, USA; Veterinary Diagnostic Laboratory, College of Veterinary Medicine, Kansas State University, Manhattan, Kansas 66502, USA; KSU Bioinformatics Center, Kansas State University, Manhattan, Kansas 66506, USA; Department of Grain Science and Industry, Kansas State University, Manhattan, Kansas 60523, USA

**Keywords:** pet food processing, canine, corn, baking, extrusion, pelleting, resistant starch, microbiome

## Abstract

Different food processing parameters may alter starch granule structure and its cooking degree. With lower thermomechanical energy, more resistant starch (RS) is retained in the food, which may benefit gastrointestinal (GI) health. The objective of this study was to determine the effect of food processing on dietary utilization and dog gut health. Experimental diets containing 56% corn as the sole starch source were produced through pelleting, baking, and extrusion and compared to a baked control diet in which the corn was replaced with dextrose. The extruded diet resulted in the highest level (*P* < 0.05) of in vitro starch cook and lowest RS, while baked was intermediate and pelleted had the lowest starch cook and highest RS. To evaluate the in vivo effects of these treatments, 12 dogs were adapted to foods for 9 d, and feces were collected for 5 d in a replicated 4 × 4 Latin square design. Feces were scored for consistency using an ordinal scale, and parametric data included apparent digestibility (ATTD), parameters indicative of gut health, and the microbial composition, which was centered log-ratio transformed before operational taxonomic unit (OTU) analyses. Fecal scores were analyzed by ordinal logistic regression, and parametric data were analyzed as mixed models. Overall ATTD was greater (*P* < 0.05) in extruded, followed by baked and pelleted. Dogs fed the control had osmotic diarrhea, whereas dogs fed the other treatments had mostly acceptable fecal scores, with extrusion leading to the best fecal quality. The control also led to high fecal pH and low SCFAs, indicating dysbiosis. All corn foods had similar (*P* > 0.05) fecal SCFAs and extruded tended (*P* = 0.055) to promote higher fecal butyrate than baked and pelleted. The microbiome of dogs fed the corn foods had similar α diversity indices, and OTUs at the species and phyla levels were mostly alike and different from the control. In conclusion, the higher levels of in vitro RS did not translate into a better in vivo fermentation profile, and extruded kibble performed best regarding fecal quality, ATTD, and fecal SCFAs.

## Teaser

Different thermal methods of processing the same-recipe food impacted in vitro resistant starch and starch fractions but did not cause significant effects on dog gut microbial activity and composition. The extruded kibble led to better fecal quality and higher apparent digestibility than the same-recipe food produced via pelleting and baking.

## Introduction

Cereals have lost popularity in the pet food industry for several reasons including suspected grain allergies and a customer perception of grains being non-nutritious fillers (Banton et al., 2021). This negative impression may have improved slightly following the July 2018 alert from the Food and Drug Administration (FDA), suggesting a link between dilated cardiomyopathy and grain-free dog diets (Mansilla et al., 2019). Some consumers may have developed the perspective that cereal grains in dog diets are unnecessary because the dog’s common ancestor, the wolf, consumed a high protein and high-fat diet from prey animals (Bosch et al., 2015). However, the domestic dog (*Canis lupus familiaris*) co-evolved with humans and has adapted to increased grain consumption (Arendt et al., 2014; Ollivier et al., 2016). As such, the dogs’ digestive system has moved closer to that of an omnivore like humans or pigs (Bosch et al., 2015).

Cereals are rich in starches, which are available sources of energy, and also contain lower amounts of dietary fiber, essential amino acids, phospholipids, and essential fatty acids that contribute to the nutritional value of the food (Corsato Alvarenga et al., 2021b; Sanderson, 2021). Starches, in particular, play an important role in food particle binding and matrix development in most processed pet foods. While highly cooked and digestible starches increase the overall digestibility of food, foods produced with lower thermomechanical energy may shift the starch nutrition from being absorbed as glucose at the small intestine (SI) to having a more compact structure that is resistant to mammalian enzymes (resistant starches [RS]; Peixoto et al., 2018; Ribeiro et al., 2019; Jackson et al., 2020; Corsato Alvarenga et al., 2021c). These RS act as prebiotics by becoming substrates for beneficial saccharolytic bacteria in the distal portions of the large intestine (Peixoto et al., 2018; Ribeiro et al., 2019; Jackson et al., 2020; Corsato Alvarenga et al., 2021c). The most relevant biomarkers of RS fermentation by the colonic microbiota of dogs are straight-chain short-chain-fatty acids (SCFA), such as butyrate (Peixoto et al., 2018; Jackson et al., 2020; Corsato Alvarenga et al., 2021c), which is the preferred energy substrate of colonocytes (Bergman, 1990) and has anti-inflammatory properties that enhance homeostasis and colonic immunity (Bedford and Gong, 2018).

Common processes used to produce animal food and feed include extrusion, pelleting, baking, canning, crumbling, and steam flaking. These processes apply different levels of moisture, time, temperature, and (or) pressure to the raw food matrix during conversion to a finished product. Pelleting is a hydrothermal process that applies a relatively small amount of steam for heat and moisture along with pressure from the die to compact the pellet and it is commonly employed in animal feed (Lancheros et al., 2020). Baking relies exclusively on thermal energy in the form of radiation, conduction, and convection. Extrusion is a continuous process that uses mechanical energy from screw shear (friction) and thermal energy from steam and water, which cook the food dough under pressure inside the extrusion barrel. Pet foods are commonly produced via extrusion, which typically promotes a high degree of gelatinized starch with starch digestibility close to 100% (Bazolli et al., 2015; Corsato Alvarenga and Aldrich, 2020). This yields very little RS in the finished product.

To increase the amount of RS in the final food, cereal processing requires approaches that protect the starch granule from mechanical shear and cooking (gelatinization), which could be through increasing particle size (Bazolli et al., 2015; Corsato Alvarenga et al., 2021d), decreasing extruder screw rotation speed (Peixoto et al., 2018; Ribeiro et al., 2019; Jackson et al., 2020; Corsato Alvarenga et al., 2021d), or increasing extruder in-barrel moisture (Baller et al., 2018; Corsato Alvarenga et al., 2021d). Most research on RS has focused on extrusion, which is the most popular process worldwide to produce kibbles. Other less common processes in the pet food industry that utilize less cooking energy, such as pelleting and baking, have been overlooked. Finding a moderate level of energy input in the dough could lead to an optimal concentration of RS remaining in the product. Thus, we hypothesized that cooking at decreasing intensities through extrusion, baking and pelleting, respectively, would lead to decreasing levels of starch cook and thereby increasing RS concentrations. The goals were to determine if processing the same recipe through different processes would lead to alternations in RS in the final food, and if higher levels of RS would translate into alterations in microbial activity and population changes in the distal gut.

## Materials and Methods

All procedures of the dog feeding study were approved by the Kansas State University institutional animal care and use committee (protocol #4097.5), in full compliance with the Animal Welfare Act and the Health Research Extension Act of 1985.

### Experimental dietary treatments

Diets for adult dogs at maintenance (AAFCO, 2022) were formulated (Concept5; Creative Formulation Concepts Staples, MN) to contain similar levels of crude protein (CP), crude fat (CF), ash, and total dietary fiber (TDF). Minor ingredient differences between the control and corn treatments were compensated by adding corn protein concentrate and adjusting the chicken meal to help achieve the target nutrient profile (Table 1). The dextrose-based negative control (control) diet was targeted to meet the same nutrient profile as the treatments without starch to and thereby contain negligible RS. The dry ration ingredients for the corn-based treatments were premixed by a commercial pet food ingredient supplier (Lortscher Animal Nutrition Inc., Bern, KS, USA). The ingredients for the control diet were sourced as individual components from the same supplier. Chicken fat (IDF, Springfield MO) and flavor digest (AFB International, St. Charles MO) were sourced before the study conduct. All foods were produced in triplicate; the baked and control were processed in three different days, whereas samples of the extruded and pelleted foods were collected at three time points during their single-day production and considered replicates.

**Table 1.** Ingredient composition (% DMB) of experimental treatments for process development of differences in RS

Ingredients	Control	Pelleted	Baked	Extruded
Corn	—	56.2	56.2	56.2
Dextrose	45.4	—	—	—
Chicken byproduct meal	14.0	13.8	13.8	13.8
Spray Dried Plasma	10.0	10.0	10.0	10.0
Corn protein concentrate	7.0	—	—	—
Fish meal	5.0	5.0	5.0	5.0
Cellulose	4.0	4.0	4.0	4.0
Dicalcium phosphate	1.75	1.00	1.00	1.00
Potassium chloride	0.65	0.40	0.40	0.40
Titanium dioxide	0.40	0.40	0.40	0.40
Calcium carbonate	0.25	0.35	0.35	0.35
Choline chloride (60% dry)	0.20	0.20	0.20	0.20
Potassium sorbate	0.10	0.10	0.10	0.10
Trace minerals premix^1^	0.10	0.10	0.10	0.10
Vitamin premix^2^	0.15	0.15	0.15	0.15
Chicken fat, internal	9.00	4.00	6.30	0.00
Chicken fat, external	1.00	3.30	1.00	7.30
Flavor	1.00	1.00	1.00	1.00

^1^Vitamin premix: vitamin E supplement, niacin supplement, thiamin mononitrate, d-calcium pantothenate, vitamin A supplement, sunflower oil, pyridoxine hydrochloride, riboflavin supplement, vitamin D3 supplement, biotin, vitamin B12 supplement, folic acid.

^2^Trace mineral premix: zinc proteinate, calcium carbonate, zinc sulfate, iron proteinate, ferrous sulfate, copper proteinate, copper sulfate, manganese proteinate, sunflower oil, sodium selenite, manganous oxide, calcium iodate, and ethylenediamine dihydroiodide.

### Food processing

#### Control

**Figure 1.**
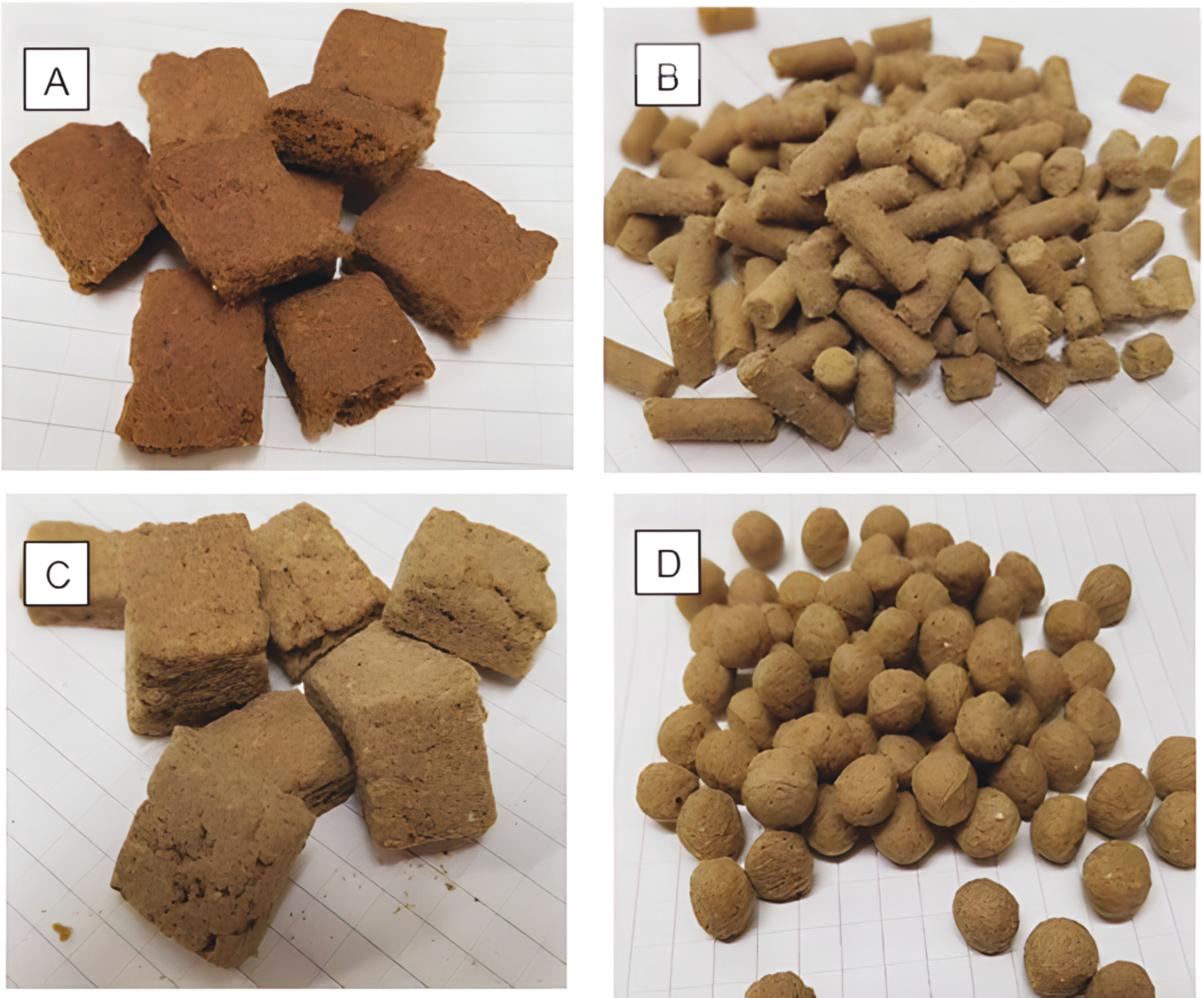
Images of the control (A) and corn-based final products (B, pelleted; C, baked; D, extruded).

Ingredients were mixed in a paddle mixer (Hobart A200, Troy Ohio) in a 4.4-kg batch size for 2 min after manually adding 9% fat. Water was added, and the dough was mixed for an additional 2 min. The dough was placed on a smooth plastic work surface and rolled into sheets to a thickness of approximately 1 cm using rolling pins and metal guides. The sheet was cut with a rotary knife into 2 cm × 2 cm squares, placed on metal cooking sheets, and cooked in a convection oven for 20 min at 150 °C. Liquid fat (1%) was coated onto the cooled squares while tumbling in a drum-style mixer for a total of 60 s. The same coating procedure was followed for the dry palatant. The final product (Figure 1A) was placed in Kraft paper polylined bags for 2 wk until the commencement of the feeding study.

#### Pelleted

Before pelleting, it was determined that the dry mix could contain up to 4% liquid fat before adversely affecting the final product. Thus, 4% liquid fat was added to the dry ingredients in a double ribbon mixer (Hayes and Stolz 23 kg HR2SSS-0106 Burleson, Texas). Once all the fat was added, the batch was mixed for 5 min. The pelleted diet was manufactured with a laboratory-scale pellet mill (CPM California Pellet Mill, model CL-5, Crawfordsville, IN) equipped with a steam injection conditioner. The downspout conditioned mash temperature was 80 °C. The die openings were 4.7 mm in diameter with a knife cutoff at 12.7 mm. Once the temperature was achieved, pellets were formed and diverted to a laboratory-scale cooler for 15 min until pellets were equilibrated to ambient conditions. During production, pellet temperature (Supplementary Table S2) was recorded by collecting pellets at the discharge and immediately placing them into an insulated container with a thermometer inserted. Other processing data recorded included flow rate (kg/min), motor load (%), and bulk density (g/L; Supplementary Table S2). The final product was coated with 3.3% chicken fat with the dry palatant. After coating, the final product (Figure 1B) was stored in bags for 2 wk until the animal feeding study was conducted.

#### Baked

Baked dietary treatment had 6.3% chicken fat included in the dry ration, which was weighed and blended into the dry mix with a paddle mixer (Hobart A200, Troy Ohio) for 2 min. A total of 1.2 kg of water was added and then mixed for another 2 min. The dough was placed onto the work surface where it was rolled to a thickness of 1 cm. Using a rotary knife, the dough was cut into 2 cm × 2 cm squares and placed on a cookie sheet. This was baked in a convection oven (Sunfire SDG-1 Cleveland, Ohio) for 25 min at 180 °C (Supplementary Table S1). The biscuits were removed and placed on racks to cool. Chicken fat with 1% added palatant was used to coat the biscuits. The final product (Figure 1C) was stored in bags for 2 wk until the commencement of the animal feeding study.

#### Extruded

The extruded diet was produced on a pilot-scale extruder (Model: X-20/E325 Wenger Mfg/Extrutech Inc., Sabetha, KS, USA) equipped with a differential diameter preconditioner with steam and water injection set to 9 and 10 kg/h, respectively. The screw profile was set to a standard pet food production setup (Supplementary Table S1). The discharge temperature out of the preconditioner was targeted at 85 °C. Processing parameters were recorded and samples were collected at three time points of production once a steady state was reached (Supplementary Table S2). Post-extrusion, kibbles were pneumatically conveyed to a double pass gas-fired pilot-scale dryer/cooler system (Wenger 4800 Series Manufacturing, Sabetha, KS) and dried at 121 °C for 5 min on the top belt, 8 min on the bottom belt, then cooled in a single pass cooler for 5 min with ambient air. The extruded kibbles were coated using a tumbling process as described previously with all the formulated chicken fat (7.3%) topically applied followed by the 1% dry palatant. The final product (Figure 1D) was then packaged into bags and stored for 2 wk until the commencement of the animal feeding study.

### Dog feeding study

Experimental diets were fed to 12 Beagle dogs (4 spayed females and 8 neutered males) of similar age (6.5 ± 0.23 yr), and body weight (BW; 11.6 ± 1.8 kg) in a replicated 4 × 4 Latin Square design. Dogs were randomly assigned to experimental treatments and periods according to the Balanced Latin Square Designed Excel spreadsheet-based program (Kim and Stein, 2009). Dogs were individually housed in pens (1.83 m × 1.20 m) with acrylic-coated mesh floors in rooms with controlled temperature (23 °C) and 16 h light/8 h dark cycle at the Large Animal Research Center (Kansas State University, Manhattan, KS, USA). Each study period consisted of 2 wk with a 9-d diet adaptation followed by a 5-d fecal collection. Each dog was fed twice daily and fresh water was provided ad libitum. Initial food amounts for each dog were calculated according to the daily energy requirements equation for inactive lab kennel dogs (NRC, 2006), and were adjusted as needed to maintain BW.

During the final 5 d of each period, total feces were collected and subjectively scored for consistency with a 5-point scale and 0.5 increments (1 = liquid/diarrhea; 2 = soft consistency, unformed stool; 3 = very moist stool that retains shape; 4 = well-formed stool that does not leave residue when picked up; 5 = very hard, dry pellets). Fecal scores of 3.5 to 4 were considered ideal. All feces were frozen at −16 °C pending further analysis. One fresh fecal sample from each dog was collected within 15 min of defecation and measured for pH by inserting a calibrated glass-electrode pH probe (FC240B, Hanna Instruments, Smithfield, RI, USA) directly into the sample. The pH was measured in three different portions of the stool and averaged. Six 2 g aliquots of fresh feces were then transferred into microcentrifuge tubes and stored at −80 °C for later analysis of SCFA and ammonia.

#### Chemical analyses

Feces were dried in a forced air oven at 55 °C for up to 48 h until moisture content was below 10%. Diets and dried fecal samples were ground using a fixed-blade laboratory hammermill (Retsch, ZM200, Haan, Germany) fitted with a 0.5-mm screen. Diets and dry feces were analyzed for dry matter and ash, and their organic matters were calculated according to methods of the Association of Official Analytical Chemists (AOAC, 2019); methods 934.01 and 942.05). Nitrogen content of the samples was determined by the Dumas combustion method using a nitrogen analyzer (FP928, LECO Corporation, Saint Joseph, MI), and crude protein was calculated using the 6.25 conversion factor (AOAC 990.03), crude fat was determined by acid hydrolysis (AOAC 954.02), and TDF was determined (AOAC 2019; method 985.29) using a TDF assay kit (TDFR-200a, Megazyme, Bray, Ireland). Gross energy was measured by bomb calorimetry (Parr 6200 Calorimeter, Parr Instrument Company, Moline, IL). Fecal marker titanium dioxide content was measured in both diets and feces according to the colorimetric method described by Myers et al. (2004). Nonstructural carbohydrate (NSC) was calculated as NSC = 100 − (Moisture + Protein + Fat + TDF + Ash). Dietary samples were analyzed for total starch (TS), rapidly digestible starch (RDS), slowly digestible starch (SDS), and RS using the digestible and resistant starch assay kit (K-DSTRS, Megazyme), and for gelatinized starch and starch cook (gelatinized starch (%) × total starch (%)) at a commercial laboratory (Wenger Manufacturing, Sabetha, KS) according to (Mason et al., 1982). The final diets’ energy absorption properties were measured by differential scanning calorimetry (DSC; Q200, TA Instruments-Waters LLC, New Castle, DE, USA). Briefly, one-part dry matter of each food and two parts water were mixed into a solution, and then 25 to 40 mg of the solution was placed into a stainless steel high-volume pan and closed with a lid that had an O-ring insertion. Each pan was inserted into the DSC, equilibrated at 10 °C, and then the temperature increased to 140 °C at 10 °C/min. Integration of the endothermic curves provided by the DSC was completed using Universal Analysis 2000 software (v. 4.7A, TA Instruments, Waters LLC).

#### Apparent total tract digestibility

Apparent total tract digestibility (ATTD) of dry matter, organic matter, crude protein, crude fat, NSC, and gross energy were determined using the titanium dioxide (TiO_2_) marker method, according to the following equations (Corsato Alvarenga et al., 2019):


$$\begin{array}{l} Nutrient\;fecal\;output(g)=\frac{\%\;nutrient\;in\;feces\times TiO2\;in\;food(g)\;}{\%\;TiO2\;in\;feces} \end{array}$$ (1)

$$\begin{array}{l} ATTD(\;\%\;)=\frac{(nutrient\;intake(g)-nutrient\;fecal\;output(g))\times 100}{nutrient\;intake(g)} \end{array}$$ (2)

The percent TiO_2_ was set as 0.40% in all foods, which was the gravimetric measure that was added during each food production.

#### Gut Health

Short-chain fatty acids (SCFAs). SCFAs were analyzed according to the protocol of Erwin et al. (1961) with the following modifications. First, fecal samples were thawed and centrifuged at 3,000 × *g* for 20 min to separate the suspended solids. A volume of 0.25 mL 25% *m*-phosphoric acid was added to 1 mL of the supernatant into a 2-mL microcentrifuge tube to acidify the sample. This was followed by a precipitation and deproteinization session at −20 °C for at least 24 h. Before analysis, deproteinized samples were thawed and centrifuged at 17,000 × *g* for 15 min and then filtered through a 0.2-μm filter into GC glass vials. Fecal SCFA contents were analyzed using gas chromatography (Hewlett-Packard 5890A, Palo Alto, CA, USA) equipped with a flame ionization detector (FID) and a column (2 m × 4 mm ID glass, packed with GP 10% SP-1200/1% H3PO4 (Supelco # 1-1965). Compressed air was set at 200 mL/min and hydrogen was set at 20 mL/min. Nitrogen was used as a carrier gas with a flow rate of 60 to 70 mL/min. The detector and injector temperatures were set at 250 °C, and column temperature at 130 °C for a total run time of 10 min. The peak area of chromatograms was determined using integrative software (GC solution version 2.42.00, Shimadzu, Kyoto, Japan). The concentrations of straight-chain SCFA (acetate, propionate, and butyrate) and branched-chain SCFA (isobutyrate, isovalerate, and valerate) in the supernatant of the fecal samples were quantified by comparing the sample peak area to a standard and correcting for the fecal DM content.

##### Microbiome

The DNA was extracted from dog fecal samples using a kit (QIAmp PowerFecal Pro DNA Kit; Qiagen, Hilden, Germany). The FASTQ files for each sample were generated and used for downstream analysis using Mothur v1.44.1(Schloss et al., 2009). The raw data were imported into the Mothur pipeline, the low-quality reads were removed, and then the 16S clean reads were aligned to the SILVA rRNA reference database (release 138; Quast et al., 2013) to assign taxonomic classification. After analysis in Mothur, an operational taxonomic unit (OTU) table and taxonomic file were generated and imported into R Studio (v4.0.3) for statistical computations (alpha and beta diversity) and data visualization. The α-diversity indices were calculated using an R package (Phyloseq; McMurdie and Holmes, 2013). The principal coordinate analysis (PCoA) figure was made using ggplot2, function “plot_ordination” in R studio. Lastly, the OTU count table defined at the genus level was processed in the following manner: absent OTUs in more than 50% of fecal samples within a treatment were removed. Then, zero counts were inflated using the Bayesian multiplicative method (Martín-Fernández et al., 2015) and converted to centered log-ratio (CLR) to normalize the arbitrary counts (generated by the DNA sequencer) of the compositional data (Aitchison, 1982; Gloor et al., 2017; Navarro-Lopez et al., 2022) before statistical analysis.

### Statistical analyses

Chemical compositions and energy of diets were analyzed with the generalized linear model (GLM) procedure from Statistical Analysis Software (SAS version 9.4, SAS Institute, Inc., Cary, NC), with diet as the fixed effect. The control, pelleted, and baked foods were analyzed in triplicates, while the extruded diet was analyzed in duplicate. Dog feeding study parametric data were analyzed as a replicated 4 × 4 Latin square design with the generalized linear mixed model (GLIMMIX) procedure of SAS v 9.4 (food intake, wet and dry fecal output, fecal DM, pH and SCFA, ATTD, and microbiome α-diversity) and JMP Pro v. 17 (microbiome OTUs), with diet and period as fixed effects and dog as the random effect. The Benjamini–Hochberg False Discovery Rate in JMP (Pro v. 17) was used to adjust treatment *P*-values of the microbiome OTUs at the species level data. Least square means were assessed using Tukey’s post hoc test for multiple comparisons. All parametric data were assessed for normality by the Shapiro–Wilk test. Fecal scores were analyzed by ordinal logistic regression with the GLIMMIX procedure with diet as the fixed effect and dog and period as random effects. The frequency of fecal scores within each diet was determined with the frequency (FREQ) procedure (SAS version 9.4, SAS Institute, Inc.). Results were considered significant at *P* < 0.05, and marginally significant at 0.05 < *P* < 0.10.

## Results

### Nutritional composition of diets

Dry matter of the extruded diet was the highest (*P* < 0.05), while pelleted and baked were intermediate, and control was the lowest just below 90% (Table 2). Although the control had a moisture >10%, what would be considered above the minimum level required for dry pet foods (AAFCO, 2022), the humectant characteristic of dextrose reduced the free water and the water activity (Aw), and all foods had an Aw below 0.5. On a dry matter basis, the protein was not equal among diets; it was the highest in baked (*P* < 0.05), intermediate in extruded, and the lowest in control and pelleted foods. Fat by acid hydrolysis had small variation but was statistically highest in control, and lowest in the baked and extruded foods. Conversely, TDF was lowest (*P* < 0.05) in control, and similar in the other treatments. The extruded food had the most gross energy than the other treatments (4,613 vs. average 4,435 kcal/g).

**Table 2.** Chemical composition of the control and corn-based pet foods produced through pelleting, baking, and extrusion (DM basis)

Item	Control	Pelleted	Baked	Extruded	*P*-value
Proximate analyses					
Dry matter, %	89.7^c^ ± 0.13	91.1^b^ ± 0.13	90.7^b^ ± 0.13	94.8^a^ ± 0.16	< 0.0001
Organic matter, %	92.5 ± 0.20	93.1 ± 0.20	92.4 ± 0.20	93.1 ± 0.24	0.0656
Ash, %	7.48 ± 0.198	6.87 ± 0.198	7.63 ± 0.198	6.86 ± 0.242	0.656
Protein, %	26.6^b^ ± 0.41	24.8^b^ ± 0.41	29.9^a^ ± 0.41	26.1^b^ ± 0.51	0.0003
Fat, %	12.8^a^ ± 0.07	12.2^b^ ± 0.07	11.9^c^ ± 0.07	12.1^bc^ ± 0.08	0.0002
TDF, %	9.60^b^ ± 0.300	10.80^ab^ ± 0.300	11.13^a^ ± 0.300	11.53^a^ ± 0.368	0.0163
Gross energy kcal/g	4,449^b^ ± 9.6	4,410^b^ ± 9.6	4,446^b^ ± 9.6	4,613^a^ ± 11.7	< 0.0001
Starch analyses					
NSC, %	33.2^bc^ ± 0.70	36.5^ab^ ± 0.70	30.3^c^ ± 0.70	38.2^a^ ± 0.86	< 0.0001
TS, %	31.0^c^ ± 0.54	40.0^a^ ± 0.54	36.3^b^ ± 0.54	40.0 ^a^ ± 0.66	< 0.0001
TDS, %	30.9^c^ ± 0.52	37.9^a^ ± 0.52	35.0^b^ ± 0.52	39.3^a^ ± 0.64	< 0.0001
Gelatinized starch, %	29.80^b^ ± 0.466	8.41^d^ ± 0.466	14.73^c^ ± 0.466	34.23^a^ ± 0.571	< 0.0001
Starch cook, %	95.4^a^ ± 1.29	19.9^d^ ± 1.29	40.3^c^ ± 1.29	86.0^b^ ± 1.57	< 0.0001
RS, %	0.086^d^ ± 0.0722	2.094^a^ ± 0.0722	1.323^b^ ± 0.0722	0.534^c^ ± 0.0884	< 0.0001
RS, % of TS	0.277^d^ ± 0.1857	5.250^a^ ± 0.1857	3.643^b^ ± 0.1857	1.342^c^ ± 0.2275	< 0.0001
RDS, %	30.2^b^ ± 0.38	13.9^d^ ± 0.38	22.1^c^ ± 0.38	35.8^a^ ± 0.46	< 0.0001
SDS, %	0.98^c^ ± 0.478	17.02^a^ ± 0.478	9.84^b^ ± 0.478	2.82^c^ ± 0.586	< 0.0001
Enthalpy, J/g	0.014^c^ ± 0.1503	3.498^a^ ± 0.1503	1.415^b^ ± 0.1503	0.013^c^ ± 0.1841	< 0.0001

NSC, nonstructural carbohydrate; TS, total starch; TDS, total digestible starch; RS, resistant starch; RDS, rapidly digestible starch; SDS, slowly digestible starch.

^abc^Means in a row with unlike superscripts differ (*P* < 0.05).

All starch parameters differed (*P* < 0.05) across dietary treatments (Table 2). Calculated NSC was highest in control, pelleted and extruded, and lower in control. Both TS and TDS were the highest in pelleted and extruded, intermediate in baked, and the lowest in control. Starch gelatinization and RS behaved as expected; the control had a starch cook close to 100% and negligible RS because no starch was present in this treatment. The pelleted, baked, and extruded foods followed an increasing order of starch cook and decreasing order of RS according to the expected energy levels added to each process type, both when measuring RS in the whole food and as a percentage of TS. Accordingly, levels of RDS increased whereas SDS concentration declined as cooking intensity increased. The enthalpy of the diets was highest for the pelleted food followed by baked due to a greater amount of uncooked starch, whereas the extruded diet was similar to the control with nearly no uncooked starch.

### Intake, fecal characteristics, and ATTD

Food intake did not differ among dogs fed the corn treatments, but was lower (*P* < 0.05) for the control food (Table 3). The control food also led to a decrease in BW (−2.2% weight change; Table 3), whereas dogs fed the extruded food had the highest weight gain. The wet fecal outputs of dogs fed the pelleted and baked diets were greater than dogs fed the control diet, and dogs fed the extruded treatment had wet fecal outputs similar to the extremes (Table 3). Similarly, after drying, there was more (*P* < 0.05) fecal dry mass when dogs were fed the pelleted, baked and extruded foods compared to the control diet (average 38.3 vs. 18.7 g/d). Fecal pH from dogs fed the control diet was the highest (6.87) among treatments (*P* < 0.05), followed by the extruded and pelleted that had similar values (average 5.7), and the baked treatment which had the lowest fecal pH measured (5.4; Table 3).

**Table 3.** Food intake, fecal characteristics, and ATTD using titanium dioxide marker method of dogs (*n* = 12) fed a control diet formulated with dextrose and experimental diets with corn produced through pelleting, baking and extrusion in a Latin square design

Item	Control	Pelleted	Baked	Extruded	SEM	*P*-value
Food intake and fecal characteristics						
Food intake, g DM/d	158.0^b^	195.0^a^	194.0^a^	204.0^a^	10.40	0.0015
Weight change, % in 14 d	−2.20^c^	2.20^ab^	1.45^b^	4.02^a^	0.623	< 0.0001
Wet fecal output^1^, g/d	70.8^b^	111.1^a^	104.7^a^	91.1^ab^	10.88	0.0005
Dry fecal output^1^ g/d	18.7^b^	40.8^a^	38.0^a^	36.1^a^	3.23	< 0.0001
Fecal DM, %	264^b^	36.7^a^	36.3^a^	39.6^a^	1.02	< 0.0001
Fecal pH	6.9^a^	5.5^bc^	5.4^c^	5.8^b^	0.10	< 0.0001
Fecal total SCFA, mol/g feces DM^2^	63.8^b^	220.3^a^	204.2^a^	235.5^a^	10.27	< 0.0001
Acetate, %	63.5^a^	51.9^b^	62.2^a^	54.2^b^	2.14	< 0.0001
Propionate, %	16.5^c^	35.8^a^	26.2^b^	30.9^ab^	1.96	< 0.0001
Butyrate, %	9.57	7.59	7.57	9.65	0.706	0.0552
Isobutyrate, %	3.55^a^	2.82^ab^	2.59^b^	3.12^ab^	0.215	0.0174
Isovalerate, %	2.46^a^	1.45^b^	1.12^b^	1.93^ab^	0.225	0.0011
ATTD						
Dry matter, %	79.7^a^	76.3^b^	77.3^b^	80.5^a^	0.64	0.0001
Organic matter, %	82.9^a^	80.6^b^	80.6^b^	84.5^a^	0.61	< 0.0001
Crude protein, %	76.8^c^	79.9^bc^	81.2^b^	85.7^a^	1.02	< 0.0001
Crude fat, %	97.4^b^	96.5^c^	97.5^ab^	98.2^a^	0.20	< 0.0001
NSC, %	95.4^b^	97.2^ab^	95.2^b^	98.4^a^	0.60	0.0010
Gross Energy, %	85.5^a^	82.9^b^	82.9^b^	86.5^a^	0.51	< 0.0001

SCFA, short-chain fatty acids; NSC, nonstructural carbohydrate.

^1^Fecal output includes all feces harvested during the collection period, but does not reflect the true output due to coprophagy and events of diarrhea or stepping on feces.

^abc^Means in a row with unlike superscripts differ *P* < 0.05.

Fecal total SCFAs were nearly four times more concentrated in all the corn-based foods relative to baked dextrose-based control (Table 3). The proportions of acetate as percentages of total SCFA were lower in the feces of dogs fed the pelleted and extruded foods compared to the other treatments, while propionate was more concentrated in dogs fed the extruded and pelleted, intermediate in baked, and lowest in control. Branched-chain SCFAs isobutyrate, isovalerate, and valerate were similar and lowest in the corn-based treatments compared to control (Table 3).

The ATTDs of diets determined by indigestible marker TiO_2_ for dogs fed corn-based foods (Table 3) were consistent with the results expected based on the starch cook data (Table 2). Dry matter, organic matter (OM), crude protein, crude fat, calculated NSC, and gross energy ATTD were lower when dogs were fed the baked and pelleted foods compared to when they were fed the extruded food (*P* < 0.05). Dogs fed the control had the lowest crude protein ATTD, but DM, OM, and gross energy ATTD were similar to the extruded, and crude fat ATTD was similar to the baked treatment.

**Figure 2.**
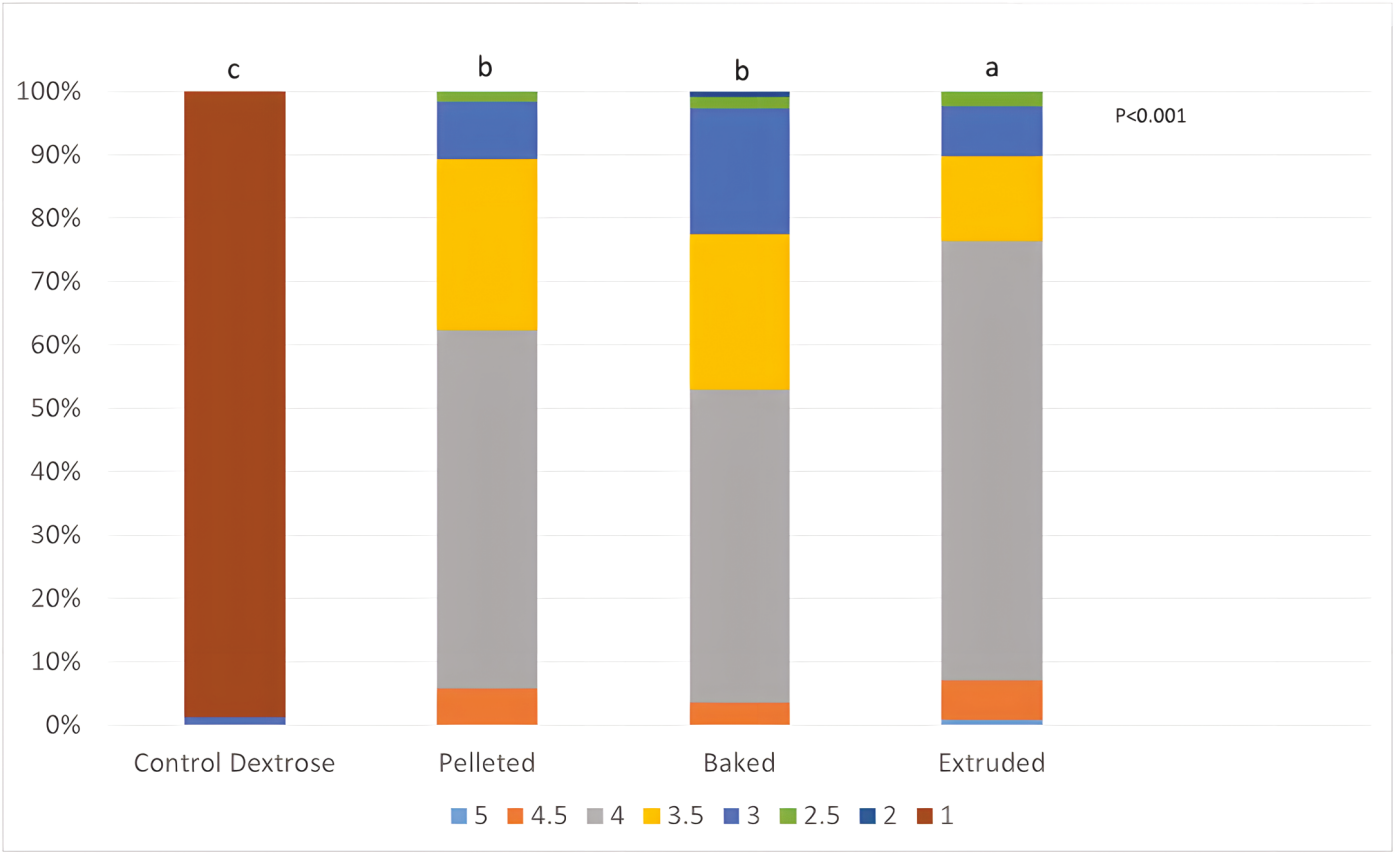
Percent distribution of fecal scores over 5 d fecal collection from dogs fed a control diet formulated with dextrose and experimental diets with corn produced through pelleting, baking and extrusion. Scores were on a 1 to 5-point scale with 0.5 increments (1 = liquid; 2 = soft, unformed stool; 3 = soft and retains shape; 4 = well-formed and consistent stool; 5 = very hard, dry feces; ideal score 3.5 and 4.0). ^abc^Fecal score among treatments with unlike letters differ *P* < 0.05.

All dogs fed the control food had diarrhea during the fecal collection phase (fecal score 1, Figure 2). This is likely the cause of their weight loss. Stool scores from dogs fed the pelleted and baked diet were similar, with most feces being consistent and firm (range of 3 to 4), whereas dogs fed the extruded had more feces scored as 4 (Figure 2). Although the extruded treatment led to more frequent ideal fecal scores, dogs fed the pelleted and baked treatments also produced acceptable feces (Figure 2).

### Microbiome

#### Alpha and beta diversity

Alpha diversity refers to variation in the microbiome community within each dog, and there were marked differences in the present study (Table 4). Observed and Chao indices are assessment metrics of the microbial species richness, and both were not different (*P* > 0.05) across treatments. The Simpson and Shannon indices account for both richness and evenness of the compositional data and these results suggested that dogs fed the control had a more diverse microbial community than dogs fed the treatment.

**Table 4.** Αlpha diversity indices of dogs (*n* = 12) fed a control diet formulated with dextrose and experimental diets with corn produced through pelleting, baking and extrusion in a Latin square design

Alpha diversity indices	Control	Pelleted	Baked	Extruded	SEM	*P*-value
Observed	236	217	222	229	11.7	0.6113
Chao^1^	1,197	1,716	1,476	1,560	147.7	0.0949
Simpson	0.944^a^	0.846^b^	0.864^b^	0.890^ab^	0.0180	0.0025
Shannon	3.57^a^	2.70^b^	2.89^b^	3.00^b^	0.114	< 0.0001

^1^One outlier removed; SEM for extruded treatment Chao was 155.3.     ^ab^Means in a row with unlike superscripts differ *P* < 0.05

**Figure 3.**
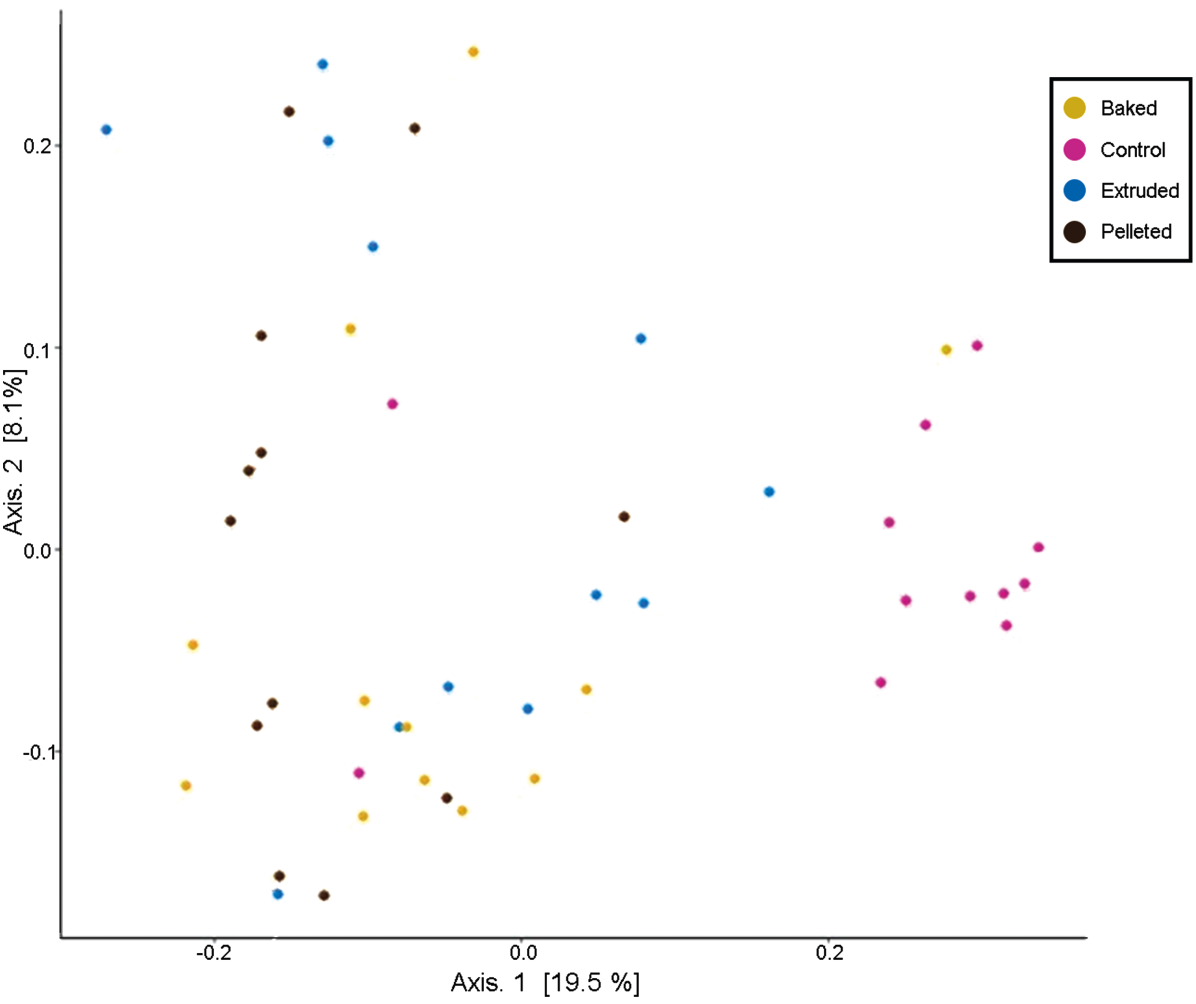
Beta diversity (principal coordinate analysis; PCoA) of the microbiome of dogs (*n* = 12) fed a control diet formulated with dextrose and experimental diets with corn produced through pelleting, baking and extrusion in a Latin square design.

Beta diversity visualized by PCoA indicated some separation of the microbiome for dogs among the four treatments. The clearest cluster belonged to the microbiome of dogs fed the control, indicating that most of these dogs had a similar microbiome (Figure 3).

#### Microbiome OTUs

**Figure 4.**
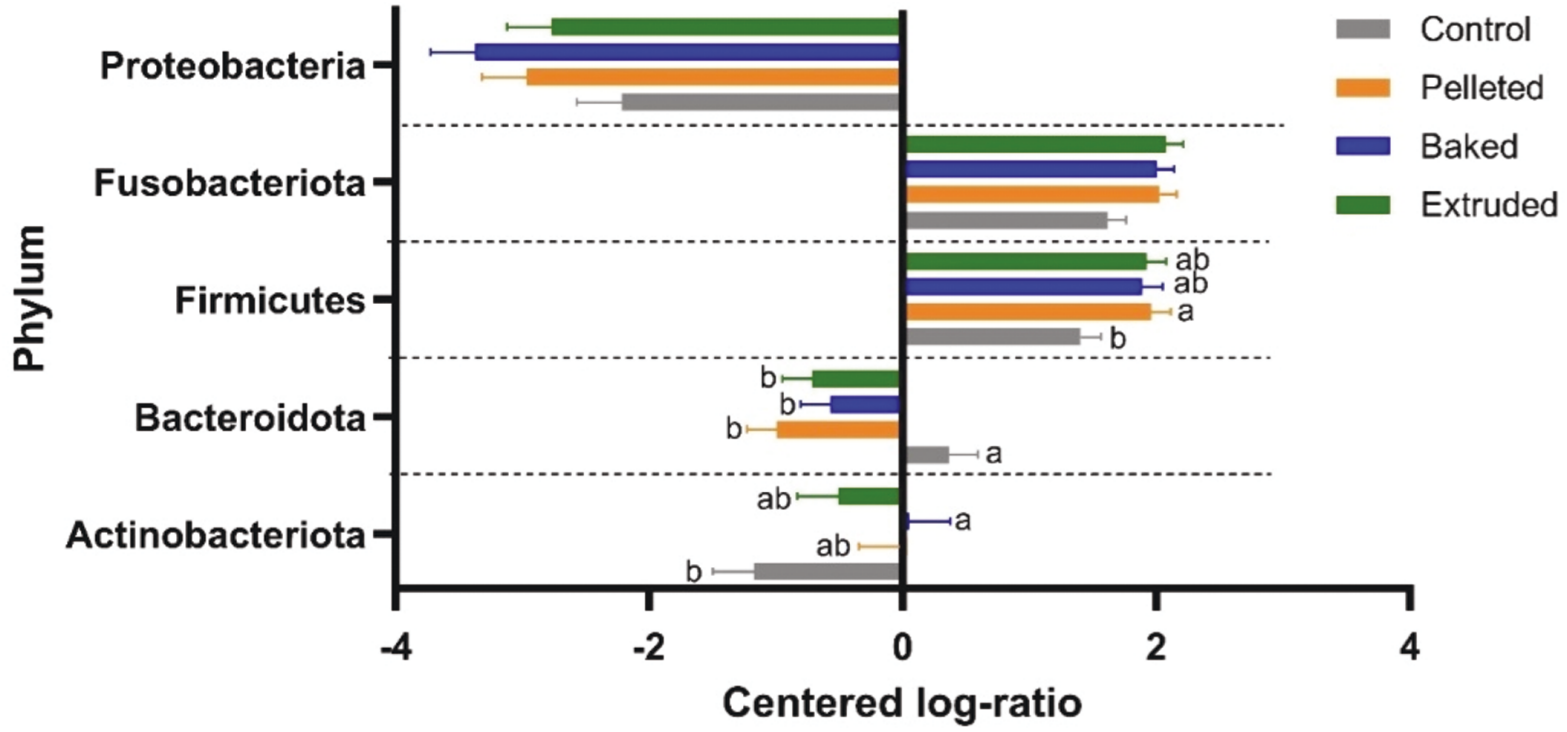
CLR of bacteria at the phylum level in feces of dogs (*n* = 12) fed a control diet formulated with dextrose and experimental diets with corn produced through pelleting, baking and extrusion in a Latin square design. *P*(treatment): Proteobacteria = 0.155; Fusobacteriota = 0.062; Firmicutes = 0.038; Bacteroidota = 0.002; Actinobacteriota = 0.049. otu25: Fusobacteriaceae Fusobacterium; otu99: Clostridiaceae Clostridiaceae; otu151: Clostridiaceae Clostridiaceae; otu48: Clostridiaceae Clostridium; otu12: Erysipelatoclostridiaceae Catenibacterium; otu63: Erysipelatoclostridiaceae Erysipelotrichaceae; otu3: Erysipelotrichaceae Turicibacter; otu20: Erysipelotrichaceae Turicibacter; otu121: Erysipelotrichaceae Turicibacter; otu9: Lachnospiraceae Blautia; otu32: Lachnospiraceae Blautia; otu71: Lachnospiraceae GCA.900066575; otu81: Lachnospiraceae Lachnoclostridium; otu124: Lachnospiraceae Lachnoclostridium; otu119: Lachnospiraceae Lachnoclostridium; otu62: Lachnospiraceae Lachnoclostridium; otu156: Lachnospiraceae Lachnoclostridium; otu57: Lachnospiraceae Lachnoclostridium; otu110: Lachnospiraceae Lachnospiraceae; otu18: Lachnospiraceae Lachnospiraceae; otu117: Lachnospiraceae Lachnospiraceae; otu104: Lachnospiraceae Lachnospiraceae; otu125: Lachnospiraceae Lachnospiraceae; otu77: Lachnospiraceae Lachnospiraceae; otu84: Lachnospiraceae Lachnospiraceae; otu93: Lachnospiraceae Lachnospiraceae; otu44: Lachnospiraceae Lachnospiraceae; otu150: Lachnospiraceae Lachnospiraceae; otu72: Lachnospiraceae Lachnospiraceae; otu15: Lachnospiraceae Lachnospiraceae; otu49: Lachnospiraceae Lachnospiraceae; otu38: Lachnospiraceae Lactonifactor; otu126: Butyricicoccaceae Butyricicoccaceae; otu91: Oscillospiraceae Colidextribacter; otu112: Oscillospiraceae Flavonifractor; otu106: Oscillospiraceae Oscillibacter; otu149: Oscillospiraceae Oscillibacter; otu52: Oscillospiraceae UCG.005; otu132: Oscillospiraceae UCG.005; otu70: Oscillospiraceae uncultured; otu17: Ruminococcaceae Faecalibacterium; otu51: Ruminococcaceae Fournierella; otu37: Ruminococcaceae Negativibacillus; otu80: Ruminococcaceae Ruminococcaceae; otu75: Ruminococcaceae uncultured; otu166: Peptostreptococcaceae Peptoclostridium; otu59: Peptostreptococcaceae Terrisporobacter; otu6: Selenomonadaceae Megamonas; otu46: Bacteroidaceae Bacteroides; otu47: Bacteroidaceae Bacteroides; otu40: Prevotellaceae Alloprevotella; otu1: Bifidobacteriaceae Bifidobacterium; otu67: Coriobacteriaceae Collinsella; otu137: Coriobacteriaceae Collinsella; otu53: Eggerthellaceae Slackia.

There were 131 OTUs in the dataset after removing those that were absent in more than 50% of dogs belonging to the same treatment. Analysis at the phylum level revealed that all treatments had a lower CLR of Proteobacteria relative to the other bacteria (Figure 4). There was a higher concentration of Fusobacteriota and Firmicutes in all treatments, and only Firmicutes were less abundant in control than pelleted. Bacteroidota were more abundant in the feces of dogs fed the control than the corn-based diets, whereas Actinobacteriota had the lowest CLR in control.

**Figure 5.**
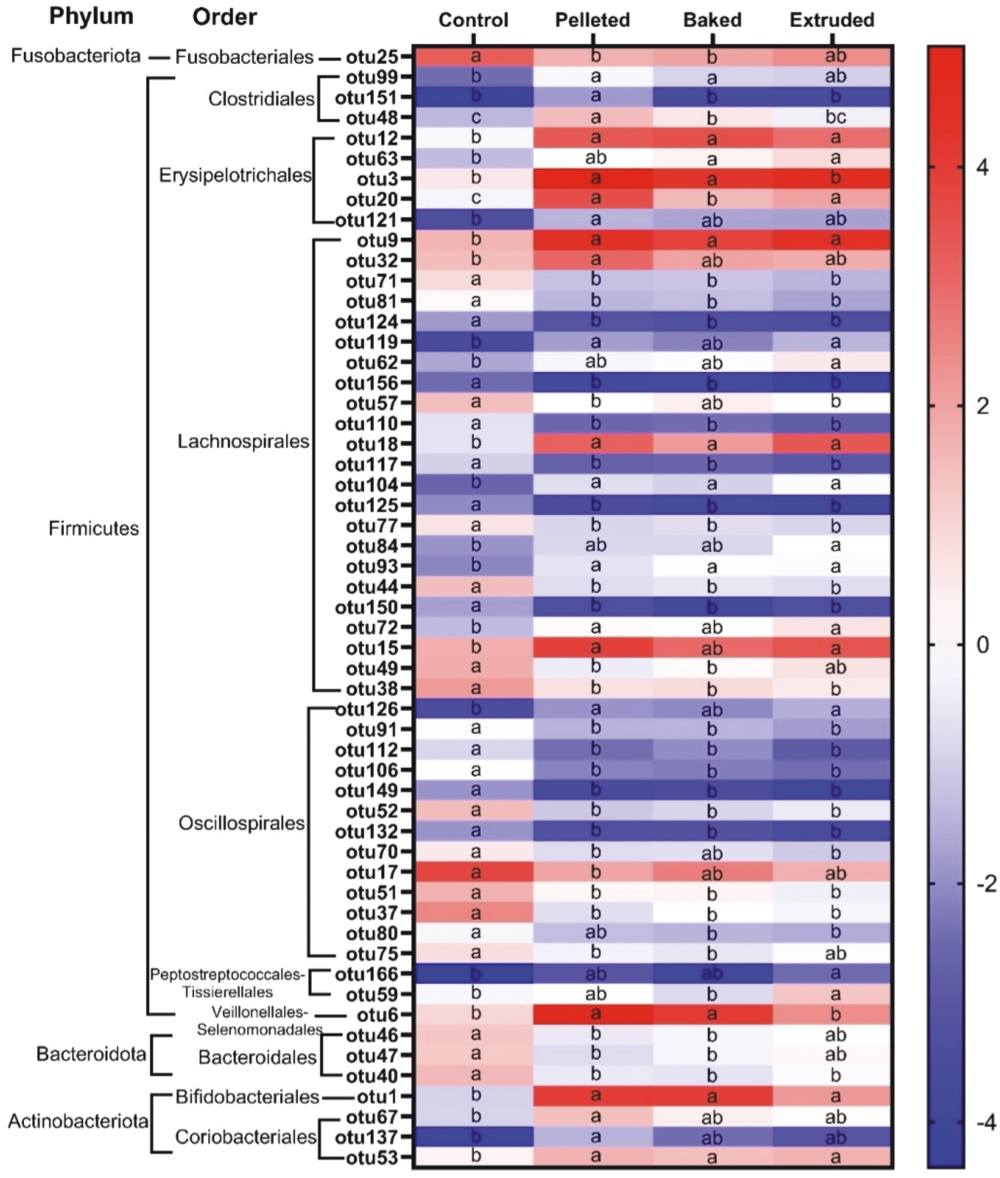
CLR of significant OTUs at species level (*P* (FDR) < 0.05) of dogs (*n* = 12) fed a control diet formulated with dextrose and experimental diets with corn produced through pelleting, baking and extrusion in a Latin square design.

Fifty-five OTUs were significant (*P*(FDR) < 0.05) at the species level (identified at the genus level), and differences were mainly driven by the control food (Figure 5): 48, 40, and 41 OTUs had different abundances when comparing the microbiome of dogs fed the control vs. pelleted, control vs. baked, and control vs. extruded, respectively. Less than three OTUs were different in the pairwise comparisons of the other treatment groups. There was one OTU of the Fusobacteriota phylum, Fusobacteriales order that was greater in control than pelleted and baked, while extruded treatment was similar to all. The Firmicutes phylum had the most changes across treatments; three Clostridiales and five Erysipelotrichales were less abundant in control vs. treatments. Within Firmicutes, the Lachnospirales order had 13 OTUs which were greater in the feces of dogs fed the control, and 10 that were greater in the treatments. In Oscillospirales order, 12 OTUs were more abundant in the feces of dogs fed the control, and only one was decreased in this treatment group compared to the others. The last OTUs in the Firmicutes phylum to have a significant change were two Peptostreptococcales-Tissierellales that were lower in the control vs. extruded food, and one Veillonellales-Selenomonadales that was more abundant in feces of dogs fed the pelleted and baked than the control and extruded foods. Three OTUs belonging to the Bacteroidota phylum, Bacteroidales order were greater in the control than the other groups. Conversely, the four OTUs belonging to the Actinobacteriota phylum were less abundant in the feces of dogs fed the baked dextrose-based control vs. corn-based treatments.

## Discussion

### Food processing

The first study goal was to determine the impact that different processing methods have on starch gelatinization, as it relates to the retention of RS in the kibble that may affect starch utilization by the dog. We hypothesized that the same-recipe pet food processed through different processes like pelleting, baking, and extrusion would yield an increasing concentration of RS and a corresponding decreasing order in gelatinized starch. As starch gelatinizes in the presence of water, it becomes water-soluble (hydroxyl groups exposed), loses birefringence, and swells (Cheftel, 1986). Gelatinized starch provides functionality to the food, making it more accessible for digestion by mammalian enzymes. In contrast, a portion of the ungelatinized starch resists digestion at the SI (RS type 2) and promotes health benefits as a prebiotic in the large intestine (Peixoto et al., 2018; Ribeiro et al., 2019; Jackson et al., 2020; Corsato Alvarenga et al., 2021c).

The first study hypothesis was confirmed by starch in vitro wet chemistry assays. Regardless of the thermal cooking process, some starch gelatinization occurs and RS present in grains decreases (Murray et al., 2001). The degree of starch transformation depends on the different processes and energy inputs which can be seen in the present study. The physical method of determining enthalpy measured via the DSC was used to determine starch gelatinization. The results reinforced those observed with enzymatic methods. The more energy (J/g) the sample absorbed corresponded to the potential for more energy applied which increased the level of starch cook and gelatinization (Pungor, 1995). These results confirm the experimental premise regarding the added cooking intensity for the treatments in the current experiment. Wherein, the pelleted and baked diets absorbed at least twofold more J/g than the extruded or control diet. This differential shows how much of an influence energy input can have on aspects of starch digestibility and conversely the level of RS. Following this same relationship for starch cook and starch gelatinized, RS declined with increased cooking intensity.

The total starch in the baked treatment was lower than the pelleted and kibble treatments, even though they were produced from the same premixed ration. No previous research was found in published literature that reported a similar outcome. To verify this difference, the samples were re-analyzed, and similar results were observed. Moreover, NSC, which is the calculated total starch, yielded similar results that confirmed the baked food had less available starch. Total starch can be affected by processing (Bernal et al., 2002), so it may be that baking led to more Maillard reaction products which made some of the starch unavailable for quantification. Although the control food had no starch. This is an artifact of the assay in which it yields a value for total starch because dextrose is α-glucose, which is the compound measured by the assay after enzymatic hydrolysis of the starch. Thus, this procedure measures glucose by colorimetry and not starch per se, so dextrose will appear as if it were starch. This factor is also why the control diet had the highest gelatinized starch and RDS values as the assay ultimately measures starch by conversion to glucose. As anticipated, the extruded kibble resulted in the highest concentration of gelatinized starch among the corn-based treatments, which agrees with what has been previously reported (Baller et al., 2018; Pacheco et al., 2018; Corsato Alvarenga et al., 2021d). Native cereal starches have concentric layers of semicrystalline layers that are ordered and compact which slows enzymatic digestion, yielding high amounts of SDS and RS in in vitro assays (Zhang et al., 2006). Extrusion imparts mechanical and thermal energy delivered via water and steam, which disrupts its crystalline structure and swells the granule, thus reducing SDS and RS and increasing RDS (Yan et al., 2019; Corsato Alvarenga et al., 2021d). Different degrees of moisture in the extruder barrel and (or) specific mechanical energy have been previously demonstrated to modify levels of RS and starch gelatinization in the final kibble (Peixoto et al., 2018; Jackson et al., 2020; Corsato Alvarenga et al., 2021d), but literature on the effects of different processing methods on starch properties in pet foods literature is lacking.

Unlike extrusion, baking relies solely on thermal energy, while pelleting utilizes a small amount of thermal energy and mechanical pressure to aid the forming of the pellets (Abdollahi et al., 2013). The steam application is primarily a source of heat that is utilized to help agglomerate food particles, which in turn reduces waste, particle segregation, denatures antinutritional factors, and thereby increases nutrient digestibility (Lancheros et al., 2020). Therefore, small amounts of gelatinization are inevitable as moisture is added (Lewis et al., 2015). İnal et al. (2018) produced a same-recipe dog food through extrusion and pelleting, and like the present study, they found that extrusion resulted in at least four times more gelatinization than pelleting. Had they (İnal et al., 2018) produced a baked biscuit, they might have seen similar results to the present study. During baking, the water is limited and the biscuit is cooked through convection, radiation, and conduction (Sakin et al., 2009). The baking oven had a higher temperature, and the residence time was five times longer compared to pelleting (Supplementary Table S2), which may explain the intermediate levels of starch gelatinization and RS.

### Dog feeding study

The second study goal was to assess the effects of baked dextrose-based control and corn-based foods produced through pelleting, baking, and extrusion on dog gut microbial activity and composition. We hypothesized that the baked food with intermediate levels of RS would lead to a fermentation profile close to ideal, while the extruded food would cause a lower impact on fermentation, and pelleted might cause overfermentation.

Starches are semicrystalline granules formed during photosynthesis and stored in various parts of the plant. They are composed of α-1,4 linked glucose polysaccharides that form linear structures of amylose (ca. 20%-30%) and larger branched amylopectin (ca. 70%-80%; Zobel, 1988; Corsato Alvarenga et al., 2021a). Mammalian enzymes can break these α-1,4 glucose bonds in digestible starches, releasing glucose which is absorbed in the SI. Several factors influence the rate and extent of starch digestion, including cell wall structure and granule morphology (i.e., cereals vs. legumes; Bhattarai et al., 2017, 2018) amylose–amylopectin ratio (Gajda et al., 2005), extent of grinding or milling (Bednar et al., 2001; Bazolli et al., 2015), and cooking parameters (Baller et al., 2018; Jackson et al., 2020; Corsato Alvarenga et al., 2021d). By limiting the starch source to whole corn and preprocessing (grinding and mixing) the raw recipe equally for the three treatments (pelleting, baking, and extrusion), the different types of processing were the only factors influencing how corn starch was utilized by the dogs.

The primary method used in the present study to characterize starch was enzymatic and focused on the rate of digestion (McCleary and Monaghan, 2002). This method measures RDS and SDS by colorimetry after specific times of incubation. RS is the undigested fraction left after 4 h of incubation with pancreatic α-amylase and fungal amyloglucosidase. This in vitro method is meant primarily for the measurement of human food starch fractions, but it was chosen because humans have similarities to canines regarding starch digestion and because there is no official method for canines. Further, by applying the same methodology to all foods one can compare all treatments relative to one another. In previous work, in vitro RS did not correlate with in vivo ileal starch digestibility of dogs (Murray et al., 1999). However, a recent study using a corn recipe produced via extrusion at three levels of thermomechanical energy found that minimal changes in RS measured using the same in vitro protocol (McCleary et al., 2002) were sufficient to positively impact markers of gut health (Corsato Alvarenga et al., 2021c). We expected similar findings in the present study. However, one needs to take into consideration that there are several physiological factors that in vitro methodologies do not account for, including actual gastric residence time, small intestine passage rate, hormones involved in the digestion process (Dhital et al., 2017), and mastication intensity (Ranawana et al., 2010) among other individual factors, which could be impacted by different processing methods (i.e., vs. all extruded kibbles at different cooking intensities; Corsato Alvarenga et al., 2021c). These differences between in vitro and in vivo digestion might help explain the differing results.

Nutrient digestibility of the corn-based diets was directly proportional to the extent of gelatinized starch in each treatment, being lower for pelleted, intermediate for baked, and highest for extrusion, as expected. Similarly, İnal et al. (2018) also reported that pelleting led to lower digestibility in dogs when compared to extrusion. This lower digestibility of the pelleted diet is likely due to the nearly raw nature of the starch in the diet using this processing method. Another study also showed that extruded dog food led to OM digestibility proportional to the level of gelatinized starch, and inversely proportional to RS (Peixoto et al., 2018). In the present study, the higher OM digestibility measured in dogs fed the extruded food, along with a lower fecal output and higher frequency of firm and ideal feces than the other corn-based treatments might indicate that extrusion could result in preferred outcomes by pet owners who have to clean-up after their pets.

It was a study concern that the pelleted food would contain high levels of uncooked starch that would lead to an overfermentation in the colon with the production of soft feces, which could potentially cause loose stools in some dogs (Goudez et al., 2011). However, fecal scores of dogs fed the pelleted and baked foods were similar and acceptable, while dogs fed the extruded food produced feces closest to the ideal, and only dogs fed the control had unacceptable stools. The frequent diarrhea in the control group was unintentional and likely happened due to large amounts of hypertonic solute (dextrose) present in the intestinal lumen, which pulled water due to differences in osmotic pressure (Marks, 2020).

The liquid stool of dogs fed the control had a higher pH and lower total SCFA in comparison to the other treatments. A high fecal pH along with low concentrations of straight-chain SCFA have been associated with putrefaction in the colon of dogs (Jackson and Jewell, 2019). In contrast, the feces of dogs fed the corn treatments had higher concentrations of straight-chain SCFA and a lower pH. The lower fecal pH may have been associated with elevated lactic acid production due to the increased RS fermentation in the colon (Peixoto et al., 2018; Ribeiro et al., 2019; Corsato Alvarenga et al., 2021c), although this was not measured. When comparing the corn-based diets, the fecal pH of the extruded treatment was higher than baked, but the total SCFAs were similar, and butyrate tended to be at a greater proportion in dogs fed the extruded food rather than pelleted or baked. Butyrate is a byproduct of glucose fermentation (Den Besten et al., 2013); it is the preferred energy source of colonocytes and has been associated with colonic cancer prevention and anti-inflammatory potential (Leonel and Alvarez-Leite, 2012). For these reasons, butyrate has been the target SCFA in animal and human studies. Acetate and propionate are other straight-chain SCFAs beneficial to the host that are known to increase with the addition of soluble fibers in pet food (Donadelli et al., 2019). While acetate can be used by various organs as fuel source, or be converted to fat, propionate is mostly converted to glucose in the liver (Bergman, 1990). One molecule of glucose will yield two acetates, two propionates, or one butyrate (Donadelli et al., 2019). Interestingly, baking induced a greater proportion of acetate, and pelleting and extrusion induced a higher proportion of propionate. Our findings were interesting; although both the pelleted and baked foods had more RS measured in vitro, the fermentation profile of the pelleted food was closest to extruded, and dogs fed the extruded food tended to produce more butyrate in the colon, which is desirable. Conversely, branched-chain SCFAs have been associated with proteolysis in the colon (Scott et al., 2013), and these were lower in the corn-based treatments compared to baked dextrose-based control. Therefore, the control showed some signs of dysbiosis.

The dog’s physiology and (or) kibble matrix likely influenced RS amounts in vivo. For instance, when measuring RS in vitro, all foods were ground using a 0.5-mm sieve size. Thus, the in vitro measurement did not account for mastication and kibble matrix. Dogs usually chew the kibbles a couple of times before swallowing, and the extrusion process develops a hard matrix as the starch gelatinizes (Donadelli et al., 2021). The pelleted and baked foods were likely more brittle, although this was not quantified. It could be that the extruded kibble’s well-formed and hard matrix along with low mastication affected how starch was digested in the SI. Whereas the weaker matrices of the pelleted and baked foods easily fragmented and exposed both cooked and raw starch to digestive enzymes at the SI. Another possibility is that extrusion was the only process that imparts mechanical shear under high pressure in the dough, which can lead to an increase in TDF and fiber solubility of the final food (Menis-Henrique et al., 2020). The extruded kibble in the present study measured higher TDF than the other treatments, so corn hemicellulose could have become more available for bacterial fermentation in the colon. These hypotheses would help explain why the fermentation profile of the pelleted and baked foods was not better than extruded, besides having more RS measured in vitro.

There are some limitations to the SCFA and microbiome analyses. Fecal SCFAs do not account for fatty acids present in proximal regions of the large intestine, nor do they account for acetate, propionate, and butyrate already absorbed in the colon by the time the fresh feces is excreted and collected. Likewise, the microbiome only accounts for the bacteria that have been excreted in the feces. When sequencing the highly preserved ribosomal 16S RNA of bacteria, the sequencer produces OTU counts that are limited to a constant sum for each sample. The data are compositional and needs to be analyzed as such (Fernandes et al., 2014). Therefore, the approach chosen in the present study was CLR, which transforms each OTU count to a natural log-ratio relative to the geometric mean of all OTUs within that given sample (Gloor et al., 2016). Some properties of CLR are that the sum of all OTUs within a sample converges to zero (the mean) and the values are scale-invariant, which means that the same ratio is expected to be obtained for a sample with few read counts and a cohort sample with many read counts (Gloor et al., 2016).

The control food was intended to be a control with no RS, and it was not intended to cause diarrhea. Consequently, the microbiome of dogs fed the control was much different from the other treatments. Two α diversity indices, Shannon and Simpson, were higher in the feces of dogs fed the control. Shannon and Simpson indices account for both richness and evenness within an individual sample, but the Simpson index places a higher weight on species evenness than species richness whereas Shannon places a higher weight on richness (Kim et al., 2017). When looking at beta diversity, the PCoA plot (Figure 5) revealed that the microbiomes among dogs fed the control were similar. When comparing the corn-based foods, results were the opposite: alpha diversity indices were smaller and like one another, and beta diversity showed some grouping among microbiomes of dogs fed each diet, but not clear clusters like the control. Hence, the individual microbiomes of dogs fed the corn-based foods did not have high variation compared to the control, but there was more inter-dog variation than the control.

Most bacterial OTUs were similar among the corn-based treatments. When comparing bacteria phylum of CLR-transformed OTUs there were more Firmicutes in pelleted than control, more Bacteroidota in control than the corn-based treatments, and more Actinobacteriota in baked than control. The tested processes, extrusion, baking, and pelleting, had similar abundances of bacteria phyla, and most of the species within these phyla were similar among the treatments. This corroborates the α diversity data which showed that the different levels of thermal and mechanical (only in extrusion) energies on corn-based foods did not have a great impact on the microbiome. The control failed as a control, as it did not behave as expected and yielded very dissonant results from the other treatments. Had the control treatment not been a part of the experiment, it might have been possible to detect small differences in the corn-based treatments as it relate to the microbiome and fermentation profile. Conversely, it demonstrated a difference that is often lacking in these studies unless there is a substantial perturbation such as the use of antibiotics (Whittemore et al., 2021).

In future studies, an alternative ingredient to replace dextrose should be considered, like a pregelatinized starch. To understand metabolic changes in dogs fed the different food types, it would also be recommended to perform metabolomics analysis in serum and feces (Corsato Alvarenga et al., 2021c; Whittemore et al., 2021). Furthermore, future studies should focus on other health benefits of feeding less cooked foods with higher concentrations of SDS and RS to dogs, including exploring the potential for controlling blood glucose levels (Ribeiro et al., 2019) and improving insulin sensitivity in obese (Maki et al., 2012) or diabetic individuals.

## Conclusion

The results of this study determined that in vitro concentrations of RDS, SDS, and RS of a corn-based recipe are affected by the processing method employed during pet food production. As the cooking intensity increased from pelleting to baking to extrusion, the RDS increased and SDS and RS decreased. Consequently, the apparent digestibility of the experimental diets increased as the cooking intensity increased. The increasing cooking intensity also affected fecal characteristics and the fermentation profile of corn-based foods in vivo; fecal scores were similar among dogs fed the pelleted and baked, and closest to ideal in those fed the extruded food; pH was lower in dogs fed the baked compared to extruded kibble, and the extruded food led to a fermentation patter similar to pelleted, but with a tendency to produce more fecal butyrate. Although there were some differences in fermentation, the microbiome diversity and compositions were similar among dogs fed the corn-based foods. Conversely, the control caused dogs to have diarrhea, with a higher fecal pH and higher proportions of branched-chain SCFA, indicating dysbiosis. In conclusion, the extruded food had less in vitro RS and SDS but performed better in vivo than the pelleted and baked foods.

## Supplementary Material

skae067_suppl_Supplementary_Table_S2

skae067_suppl_Supplementary_Table_S1

## Abbreviations

SI: small intestine
RS: resistant starch
CP: crude protein
CF: crude fat
TDF: total dietary fiber
BW: body weight
SCFA: short-chain fatty acids
TS: total starch
RDS: rapidly digestible starch
SDS: slowly digestible starch
DSC: differential scanning calorimetry
ATTD: apparent total tract digestibility
TiO_2_: titanium dioxide
OTU: operational taxonomic unit
CLR: centered log-ratio
DM: dry matter
OM: organic matter
PCoA: principal coordinate analysis
