# Supplementary material for: Processing of corn-based dog foods through pelleting, baking and extrusion and their effect on apparent total tract digestibility and colonic health of adult dogs
Source: J Anim Sci. 2024 Mar 30;102:skae067. doi: 10.1093/jas/skae067 (PMC11005766; doi:10.1093/jas/skae067)
Supplement: skae067_suppl_Supplementary_Table_S2 [file skae067_suppl_supplementary_table_s2.docx]

Table S.2. Processing parameter averages from production of dextrose control and experimental corn diets

|  | Control | Pelleted | Baked | Extruded | SEM | *P*-value |
| --- | --- | --- | --- | --- | --- | --- |
| Feed Rate, kg/hr | *NM | 1.4 | *NM | 80.8 | **N/A | **N/A |
| Process Temp, °C | 148.9^a^ | 82.8^b^ | 176.6^c^ | 100.2^d^ | 0.6 | <.0001 |
| Residence Time, min | 20.0 | 0.5 | 25.0 | *NM | **N/A | **N/A |
| Density, g/L | 424.0^a^ | 606.2^b^ | 498.0^c^ | 310.6^d^ | 6.9 | <.0001 |
| Final Product Flow Rate, kg/h | *NM | 36 | *NM | 102 | **N/A | **N/A |
| Operating torque, % | *NM | 57.0 | *NM | 44.0 | **N/A | **N/A |
| Discharge T, °C | *NM | 82.2 | *NM | 95 | **N/A | **N/A |

*NM = Not Measured

**N/A = Not Available

^abc^ Unlike superscripts differ (P<0.05)
