# Supplementary material for: Processing of corn-based dog foods through pelleting, baking and extrusion and their effect on apparent total tract digestibility and colonic health of adult dogs
Source: J Anim Sci. 2024 Mar 30;102:skae067. doi: 10.1093/jas/skae067 (PMC11005766; doi:10.1093/jas/skae067)

Table S.1. Extruder screw configuration used to produce the extruded corn-based treatment.

|  | **Screw Profile** |  |  |  | **Head Profile** |  |  |
| --- | --- | --- | --- | --- | --- | --- | --- |
| Screw number | Screw Element & Description | Length, mm |  | Head Position | Head type | Length, mm | Heat Exchange |
| 1 | Inlet, single flight, full pitch screw | 236 |  | Head 1 | Inlet smooth | 238 | Steam/Water |
| 2 | Single flight, full pitch screw | 98.5 |  | Head 2 | Center Spiral | 117.5 | Steam/Water |
| 3 | Small steam lock | 19 |  | Head 3 | Center Spiral | 117.5 | Steam/Water |
| 4 | Single flight, full pitch screw | 98.5 |  | Head 4 | Center Spiral | 117.5 | Steam/Water |
| 5 | Small steam lock | 19 |  | Head 5 | Center Spiral | 117.5 | Steam/Water |
| 6 | Single flight, full pitch screw | 98.5 |  | Head 6 | Cone Spiral | 117.5 | Steam/Water |
| 7 | Medium steam lock | 19 |  |  |  |  |  |
| 8 | Double flight, ½ pitch screw | 98.5 |  |  | **Knife profile** |  |  |
| 9 | Large Steam Lock | 19 |  | Short, hard, 6 blades | |  |  |
| 10 | Double flight,1/2 pitch, cut cone screw | 119.5 |  |  |  |  |  |
|  | Total Screw Length: | 825.5 |  |  | | | |


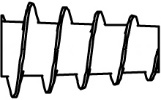

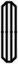

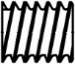

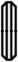

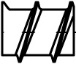

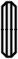

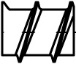

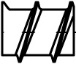

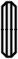

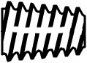

Supplement: skae067_suppl_Supplementary_Table_S1 [file skae067_suppl_supplementary_table_s1.doc]
